# Pupillary response test as a potential early diagnosis biomarker of Alzheimer’s disease

**DOI:** 10.1590/1980-5764-DN-2024-0153

**Published:** 2025-05-26

**Authors:** Robert Shen, Yuda Turana, Yvonne Suzy Handajani, Lukas, Nelly Tina Widjaja, Nova Eka Budiyanta, Reiner Adi Putra Sumarta, Celine Gabriella Wahyudi, Hans Timothy Wijaya, Jimmy Fransisco Abadinta Barus, Linda Suryakusuma, Poppy Kristina Sasmita, Tara Puspitarini Sani

**Affiliations:** 1 Atma Jaya Catholic University of Indonesia, School of Medicine and Health Sciences, Master’s Program in Biomedical Sciences, Atma Jaya Neuroscience Research, North Jakarta, Indonesia.; 2 Atma Jaya Catholic University of Indonesia, School of Medicine and Health Sciences, Department of Neurology, Atma Jaya Neuroscience and Cognitive Center, North Jakarta, Indonesia.; 3 Atma Jaya Catholic University of Indonesia, Center of Health Research, North Jakarta, Indonesia.; 4 Atma Jaya Catholic University of Indonesia, Faculty of Engineering, Cognitive Engineering Research Group, South Tangerang, Indonesia.; 5 Atma Jaya Catholic University of Indonesia, School of Medicine and Health Sciences, Department of Anatomy, North Jakarta, Indonesia.

**Keywords:** Alzheimer Disease, Biomarkers, Dementia, Doença de Alzheimer, Biomarcadores, Demência

## Abstract

Psychometric tests as the gold-standard for diagnosing Alzheimer’s disease (AD) tend to be inefficient, nonreproducible, and are affected by many factors. Research on pupillary response in AD is not novel; however, there has not been a single study providing the diagnostic value of pupillary response parameters as a recommendation for their potential use for early AD diagnosis.

**Objective:**

To investigate pupillary response parameters as clinical biomarkers of AD that are dynamic, affordable, efficient, and objective (non-operator dependent).

**Methods:**

A cross-sectional study on pupillary response parameters was conducted, with comparative and correlation-analytical tests between pupillary response and the Mini-Mental State Examination cognitive score. The diagnostic value of pupillary response parameters was obtained by determining the cutoff point of each parameter by calculating the sensitivity, specificity, Youden’s maximum index, Area Under the Curve (AUC) value from the Receiver Operating Characteristic (ROC) curve, positive predictive value, and negative predictive value. Pupillary response parameters were classified into four types: size, response time, velocity, and acceleration.

**Results:**

There were 128 respondents in total, consisting of cognitively-unimpaired young adults, cognitively-unimpaired older adults, and cognitively-impaired older adults. The pupillary response values without involving acceleration parameters were reliable. The diagnostic value of change in pupil size, the maximum and the average constriction velocity parameters have normal cognitive cutoff scores of 1.77 mm (sensitivity: 61.3%; specificity: 80%; positive predictive value [PPV]: 47.76%; negative predictive value [NPV]: 86.88%), ≥4.43 mm/s (sensitivity: 70.4%; specificity: 58.5%; PPV: 46.93%; NPV: 78.48%), and 3.12 mm/s (sensitivity: 70.4%; specificity: 55.0%; PPV: 45.83%; NPV: 77.5%), respectively.

**Conclusions:**

Pupillary response, in particular changes in constriction and velocity, can be potentially used as an early diagnostic biomarker for AD.

## RESUMO

Testes psicométricos, considerados o padrão-ouro para diagnosticar a doença de Alzheimer, tendem a ser ineficientes, não-reproduzíveis e são afetados por muitos fatores. Pesquisas sobre o exame do reflexo pupilar na doença de Alzheimer não são inéditas; no entanto, não há estudos que forneçam o valor diagnóstico dos parâmetros do reflexo pupilar como uma recomendação para o seu uso potencial no diagnóstico precoce da doença de Alzheimer.

**Objetivo:**

Investigar parâmetros do reflexo pupilar como biomarcadores clínicos da doença de Alzheimer que sejam dinâmicos, acessíveis, eficientes e objetivos (não dependentes do operador)

**Métodos:**

Foi realizado um estudo transversal sobre parâmetros do reflexo pupilar, com teste comparativo e teste analítico de correlação entre reflexo pupilar e escore cognitivo do Mini Exame do Estado Mental. O valor diagnóstico dos parâmetros do reflexo pupilar foi obtido determinando o ponto de corte de cada parâmetro pelo cálculo da sensibilidade, especificidade, índice máximo de Youden, valor de Área sob a Curva (AUC) da curva ROC (Receiver Operating Characteristic), valor preditivo positivo e valor preditivo negativo. Os parâmetros do reflexo pupilar foram classificados em quatro tipos: tamanho, tempo de resposta, velocidade e aceleração.

**Resultados:**

128 indivíduos foram entrevistados no total, sendo adultos jovens sem comprometimento cognitivo, idosos sem comprometimento cognitivo e idosos com comprometimento cognitivo. Os valores de reflexo pupilar sem envolvimento de parâmetros de aceleração são confiáveis. O valor diagnóstico de alteração no tamanho da pupila, parâmetros de velocidade de constrição máxima e média têm pontos de corte cognitivos normais de 1,77 mm (sensibilidade: 61,3%; especificidade: 80%; valor preditivo positivo [VPP]: 47,76%; valor preditivo negativo [VPN]: 86,88%), ≥4,43 mm/s (sensibilidade: 70,4%; especificidade: 58,5%; VPP: 46,93%; VPN: 78,48%) e 3,12 mm/s (sensibilidade: 70,4%; especificidade: 55,0%; VPP: 45,83%; VPN: 77,5%), respectivamente.

**Conclusões:**

O reflexo pupilar, particularmente alterações na constrição e velocidade, tem potencial para ser utilizado como um biomarcador de diagnóstico precoce para a doença de Alzheimer.

## INTRODUCTION

 Cognitive impairment, a common problem found in older adults, has the potential to progress to ward the pathological continuum of dementia, with Alzheimer’s dementia (AD) being the most common type of dementia and the 5th leading cause of death globally^1, 2^. AD cases are estimated to experience a threefold increase in trend by 2050 with high acceler ation after the COVID-19 pandemic^3^ . Mild Cognitive Impairment (MCI) progresses to the dementia stage at a rate of 7.1% per year^4^ . However, while accurate early detection is essential in suppressing the rate of progression and increasing the incidence of dementia, there are still many limitations in the diagnosis stage of the dementia continuum, which still relies on cog nitive psychometric tests as the current standard for dementia screening tools in routine clinical practice ^2, 5 , 6^ . These limitations include:

- Low reproducibility and operator-dependent;
- Time-;consuming;
- Causing psychological stress for older adults;
- Being influenced by level of education;
- Having a ceiling and floor effects even after ad justing it for level of education; and
- The potential for false positive diagnoses in pa tients with visual impairments and limitation in reading, writing, listening, and understand ing instructions ^7-11^.

The diagnoses of dementia and MCI have been evaluated over time to achieve a better definition that describes the initial pathological process and predicts risks before clinical manifestations appear ^12 ,13^. Current research and development in the early diag nostic biomarker are some of the main focuses of the global response to dementia, which is still in the early stage ^14, 15^ . Authors of many reports have previously Pupillary response test for Alzheimer’s diagnosis. Shen R, et al. discussed and developed the need for clinical diagnos tic biomarkers of AD to complement the limitations of the current screening tools, but still need analysis and improvement in terms of accuracy and effective ness. Turana et al. found that the change in pupil size to tropicamide eye drops as measured by dynamic pupillometer is beneficial for the early detection of amnestic MCI; however, tropicamide drops tend to be time-consuming and a bit uncomfortable for the patient ^16^ . As a continuation of the study, we analyzed the difference in pupillary response parameters (size, amplitude, response time, velocity, and acceleration) in the group with and without cognitive impairment and between older adults and young adults. 

 Alterations in pupillary response are observed in the neuropathological progression of AD, both clinically and histologically, due to the downregulation of excit atory and inhibitory postsynaptic receptors, impaired neurotransmitter release, and disruption of the locus coeruleus-norepinephrine (LC-NE) system’s projections to relevant brain regions ^16- 21^ . Our review of previous studies showed that the majority involved relatively small sample sizes and reported inconsistent findings regarding pupillary response in AD. Nevertheless, authors of most studies suggested a general decline in pupillary response correlating with decreasing cognitive scores ^18, 19 , 22- 28^ . While research into pupillary response in AD is not novel, to the best of our knowledge, no study has thoroughly assessed the diagnostic value of specific pupillary response parameters for the early screening of AD. Thus, we aim to investigate the diagnostic utility of these parameters, including establishing cutoff values for those significantly correlated with cognitive scores. This study could serve as a valuable reference for the early diagnosis of AD, using pupillary response measure ment as a non-psychometric and operator-independent clinical biomarker. 

## METHODS

 This cross-sectional study was conducted with a comparative analytic design in three groups:

- Cognitively-unimpaired young adults;
- Cognitively-unimpaired older adults;
- Cognitively-impaired older adults.

The sample of all young adult participants was composed of medical students, while the sample of older adult participants was collected in Kali Anyar, West Jakarta, Atma, whose cognitive status and examination was registered in the dementia study carried out by the Center of Health Research of the Atma Jaya Catholic University of Indonesia (AJCUI). Inclusion criteria for the young adult group were aging 40 years, with no history of diagnosed cognitive impairment of any etiology, and having normal global cognitive scores. The inclusion criteria for the older adult group were aging 60 years, being able to read and write, and having a history of previous cognitive status. Exclusion criteria were not being able to hear/communicate/understand instructions, not coordinating when collecting data, refusing to measure pupillary response using a dynamic pupillometer, mature cataracts, wearing patterned contact lenses, blindness (no light perception) based on neurological examination, and failure to analyze the recorded pupillary responses. The assessment processes were blinded with two independent raters for pupillary response and the Mini-Mental State Examination (MMSE) assessment. 

 Ethical approval was obtained from the Institutional Review Board (IRB) Ethical Clearance Commission, School of Medicine and Health Sciences, AJCUI, registered under the number: 12/12/KEP-FKIKUAJ/2021. Informed consent was obtained from each participant. 

### Pupillary response

 Pupillary responses recording and measurement were conducted using a dynamic pupillometer hardware and the ALZEYEMER (Alzheimer Eye-Biomarker Meter) software developed and validated by the Cognitive Engineering Research Group (CERG), Faculty of Engineering of AJCUI. The dynamic pupillometer hardware is in the form of goggles consisting of components of a full high definition (HD) 1080 pixel, 30 frames per second webcam, non-visible infrared (IR) LED light with a wavelength of 940 nm, a white visible LED light with a wavelength of 400-750 nm, and a VL530L0X proximity sensor. The desktop software operates with an Intel® Core™ i5 laptop, 8.00 gigabytes of RAM, 64-bit. 

 The pupillary response recording process is carried out for ten seconds with the following mechanism:

- IR LED lights are up throughout the measurement, with the first two seconds for the basic pupil size measurement;
- The next six seconds consist in a light intervention on the pupil using a visible white LED light to measure the time parameters, maximum constricted pupil size, pupil size change, velocity parameters, and acceleration parameters;
- The last two seconds of the intervention ended with the visible white LED light being off and the pupil returning to baseline.

**Figure 1:**
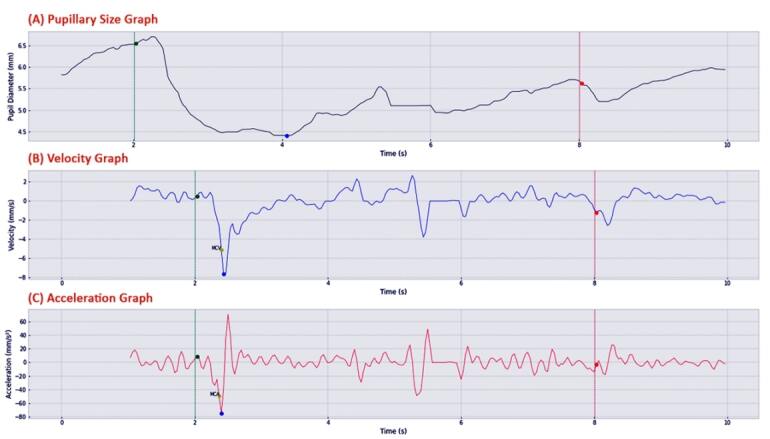
The measurement of pupillary response parameters using the ALZEYEMER software resulted in three graphs. The measurement graph starts and ends as the IR-LED lights are up and off for ten seconds. The green line indicates the beginning of the white LED light intervention, which lasts six seconds, and the red line indicates the end of the intervention. (A) The pupillary size graph shows D1 (green dot), D2 (blue dot), and the difference between D2 and D1 is calculated as ΔD. The range between the green line and the green dot is LT, while the range between the green dot and the blue dot is CT. (B) The velocity graph shows MCV (yellow dot), and ACV is calculated based on average velocity data between the green dot and the blue dot. (C) The acceleration graph shows MCA (yellow dot), and ACA is calculated based on average acceleration data between the green dot and the blue dot.

The analysis of the pupillary responses (Figure 1)consisted of nine measurement parameters that were classified into four types:

- Size parameters: baseline pupillary diameter/ D1 (in millimeter, mm), minimum constriction pupil diameter/D2 (in mm), and change in pupil size from baseline to minimum constriction pupil diameter/ΔD (in mm);
- Time parameters: latency time/LT (in seconds, s) and constriction time/CT (in s);
- Velocity parameters: maximum constriction velocity/MCV (in mm/s) and average constriction velocity/ACV (in mm/s);
- Acceleration parameters: maximum constriction acceleration/MCA (in mm/s^2^) and average constriction acceleration/ACA (in mm/s^2^ ).

## Cognitive assessment

 Cognitive assessment was done using the MMSE, which has a maximum score of 30, with a value of 24–30 indicating no cognitive impairment and a value <24 indicating cognitive impairment ^29, 30^ . This study used the validated MMSE Indonesian language version adapted by Hogervorst et al. ^29^ . The older adults participating in this study (cognitively-unimpaired and cognitively -impaired) were collected from the medical record database in the previous dementia study of the Center of Health Research of AJCUI ^31^ . The MMSE score for this group consists in is the latest data on cognitive examination carried out simultaneously with pupillary responses test data collection during the study period. 

## Statistical analysis

 Statistical analysis was done using the STATA 14 software (StataCorp LLC., Texas, USA), with a <0.005 level of significance. Bivariate analysis using the Kruskal–Wallis test was carried out to compare the pupillary response parameters and the MMSE score between each group. The diagnostic value analysis was done in each pupillary response parameter, which was reliable based on the Cronbach’s Alpha test, valid based on the validity test, and significant in the comparative analysis. Diagnostic values included:

- The cutoff value of each parameter that has the best sensitivity (Sv) and specificity (Sp) through the maximum Youden’s index;
- The area under the curve (AUC) value through the receiver operating characteristic (ROC) curve;
- Positive predictive value (PPV) and negative predictive value (NPV).

## RESULTS

 There were 141 participants involved in this study, with 128 participants meeting the inclusion criteria. A total of 13 participants were excluded; four older adults refused to be examined using the pupillometer hardware and nine participants were excluded due to: failure to detect the pupil, caused by wearing patterned contact lenses (one adult); very slanted eyes, with only a semi-circle recorded pupil (one adult); mature cataracts (three older adults); and four older adults were uncooperative, as they kept blinking during the pupillary recording process. According to demographic status data (Table 1), most participants were women (67.97%) and most older adults had level of education of <9 years (50.78%), while the young adult group, overall, had a level of education of >9 years (39.84%). 

**Table 1:** Demographic status and cognitive status of research participants.

Variables		n (%) Total	n (%) Cognitively-unimpaired young adults	n (%) Cognitively-unimpaired older adults	n (%) Cognitively-impaire older adults (MCI or AD)
Age group (years)	Young adults (≤40)	51 (39.84)	51 (39.84)	-	-
	Older adults (≥60)	77 (60.16)	-	37 (28.91)	40 (31.25)
Sex	Men	41 (32.03)	14 (10.93)	19 (14.84)	8 (6.25)
	Women	87 (67.97)	37 (28.90)	18 (14.06)	32 (25.00)
Years of education	<9 years	65 (50.78)	0 (0)	27 (21.09)	38 (29.68)
	≥9 years	63 (49.22)	51 (39.84)	10 (7.81)	2 (1.56)
Cognitive group	Cognitively-unimpaired (MMSE 24-30)	88 (68.75)	51 (39.84)	37 (28.91)	-
	Cognitively-impaired (MMSE 0-23)	40 (31.25)	-	-	40 (31.25)

Abbreviation: MMSE, Mini-Mental State Examination; n, Number.

 In the Cronbach’s Alpha reliability test, the acceleration parameter experienced a large decrease in alpha value, and the overall pupillary response parameters proved to be reliable with an overall alpha value of 0.6 and an average alpha value of 0.7396 after excluding ACA and MCA parameters (Table 2). The validity test was done by comparing results from the Pearson’s product-moment correlation table (with formula Df [degrees of freedom]=n–2), obtained at Df=126, having r=0.174; at this stage, the pupillary response parameters are proved to be valid on D1, ΔD, CT, MCV, and ACV with an item-rest correlation exceeding 0.174. 

**Table 2:** Reliability, validity, and comparative analytic test of pupillary response parameters and MMSE score for each cognitive group.

	Cronbach’s alpha*	Item-rest correlation	Median (Minimum–Maximum)			p-value Kruskal-Wallis
			Cognitively-unimpaired young adults	Cognitively-unimpaired older adults	Cognitively-impaired older adults (MCI or AD)	
D1	0.6328	0.7443	6.150 (3.427-7.568)	5.445 (2.897-6.778)	5.032 (3.350-7.171)	0.0001
D2	0.7708	0.1016	3.566 (2.070-6.287)	3.815 (2.343-4.900)	3.588 (2.420-5.545)	0.8826
ΔD	0.6619	0.6882	2.335 (1.057-4.648)	1.510 (0.176-2.883)	1.325 (0.510-2.742)	0.0001
LT	0.7583	0.1480	0.267 (0.067-0.700)	0.267 (0.133-0.667)	0.300 (0.167-1.700)	0.0761
CT	0.7687	0.4714	0.167 (0.067-0.367)	0.167 (0.067-0.267)	0.200 (0.100-0.467)	0.1112
MCV	0.6408	0.7374	5.690 (3.529-10.210)	4.161 (1.016-6.988)	4.089 (1.862-19.454)	0.0001
ACV	0.6188	0.7562	4.024 (2.046-9.633)	3.077 (1.012-4.382)	3.0715 (1.636-13.041)	0.0001
MCA	Excluded (alpha<0.6)		43.560 (14.597-124.401)	26.604 (4.536-26.360)	27.299 (8.322-115.034)	0.0001
ACA	Excluded (alpha < 0.6)		27.014 (9.496-66.273)	15.411 (3.369-36.603)	16.4965 (3.962-103.907)	0.0001
MMSE	-	-	30	27 (24-30)	19.5 (9-23)	0.0001

Abbreviation: D1, Baseline pupillary diameter; D2, Minimum constriction pupil diameter; ΔD, Change in pupil size from baseline to minimum constriction; LT, Latency time; CT, Constriction time; MCV, Maximum constriction velocity; ACV, Average constriction velocity; MCA, Maximum constriction acceleration; ACA, Average constriction acceleration; MMSE, Mini-Mental State Examination.

 According to the Kruskal-Wallis test (Table 2), the MMSE score and pupillary response parameters, except for D1 and LT, had a statistically significant relationship (p<0.05) based on cognitive groups. It can be concluded that cognitive status is related to the pupillary response of D1, ΔD, CT, MCV, ACV, MCA, and ACA. All pupillary response parameters have an abnormal distribution based on the normality test of the Shapiro-Wilk test (p<0.05). Hence, the data showed median values and minimum to maximum ranges. The cognitively-unimpaired young adult group has values of D1, ΔD, MCV, ACV, MCA, and ACA, which tend to be higher than the older adult group, whereas the older adult group with cognitively-unimpaired or cognitively-impaired individuals does not greatly differ. We concluded that the cognitive status based on the older age group tends to decrease the values of the pupil response variables. 

**Figure 2:**
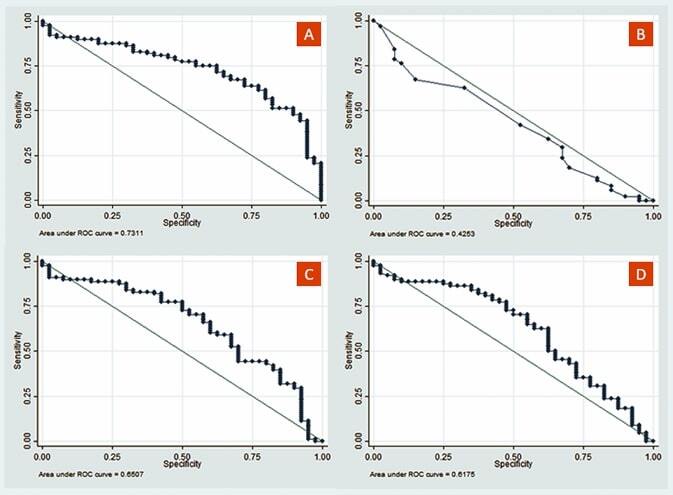
The ROC curve of ΔD (A), CT (B), MCV (C), and ACV (D) in cognitively-unimpaired and cognitively-impaired (MCI or AD) groups.

 The diagnostic value analysis was performed based on pupillary response parameters that were reliable, valid, and significantly related to cognitive status: ΔD, CT, MCV, and ACV. In the pupil size parameter, ΔD represents the value of D1 and D2 because it is a change in pupil size. The cutoff determination is necessary to classify the pupillary response parameters in the form of numerical data into categorical data to distinguish the limit values of the cognitively-unimpaired group from the cognitively-impaired group (MCI or AD), which is calculated through the ROC curve (Figure 2). The results differed between groups: the cognitively-unimpaired group had a cutoff point of ΔD 1.77 mm, MCV 4.43 mm/s, and ACV 3.12 mm/s, while CT had no diagnostic value because the AUC was below the 45% line (Figure 2B). 

 According to our findings (Table 3), pupillary response parameters of ΔD, MCV, and ACV had a relatively high diagnostic value based on the AUC-ROC curve. The overall NPV value, which is greater than PPV, indicates that the pupillary response diagnostic ability can better expect that the examined subject does not have cognitive impairment if the results of the ΔD, MCV, and ACV examinations are within normal cutoff points. 

**Table 3:** Diagnostic value of selected pupillary response parameters.

	Participants (n)		Normal cutoff value (% ROC)	Sv (%)	Sp (%)	Maximum Youden’s Index	PPV (%)	NPV (%)	OR (95%CI)
	Cognitively-impaired	Cognitively-unimpaired							
ΔD	Impaired	32	≥1.77 mm (73.11)	61.3	80.0	0.413	47.76	86.88	6.05 (0.64–0.82)
	Normal	8							
MCV	Impaired	23	≥4.43 mm/s (65.07)	70.4	58.5	0.289	46.93	78.48	3.23 (0.55–0.75)
	Normal	17							
ACV	Impaired	22	≥3.12 mm/s (61.75)	70.4	55.0	0.254	45.83	77.5	2.91 (0.51–0.73)
	Normal	18							

ΔD, Change in pupil size from baseline to minimum constriction; MCV, Maximum constriction velocity; ACV, Average constriction velocity; Sv, Sensitivity; Sp, Specificity; PPV, Positive predictive value; NPV, Negative predictive value; OR, Odds ratio; 95%CI: 95% confidence interval.

## DISCUSSION

 In this study we found that the median value and the minimum-maximum range of D1, ΔD, velocity, and acceleration parameters in cognitively-unimpaired young adults tend to be higher than in the older adult groups, reflecting a decrease in pupillary response in the older adults. Meanwhile, smaller CT values in cognitively-unimpaired adults indicate less time to reach maximum constriction. This finding is similar to the literature review on pupillary response in dementia participants conducted by Chougule et al., who concluded that although there were variations in several studies, the cognitive decline in dementia tended to decrease the amplitude, velocity, and acceleration of maximum pupillary constriction ^32^ . Variation in the findings of several studies is due to the relatively small number of research participants and the different age groups of the participants ^32^ . 

 Nevertheless, in this study, involving 77 older adult participants, we found relatively small changes in pupillary response in the cognitively-unimpaired and the cognitively-impaired groups of older adults. According to our analysis, the other studies only involved the dementia group, whereas in this study we included older adults with MCI and AD into one group based on cognitive decline according to the MMSE scoring, which has various limitations in terms of precision and specificity for defining cognitive groups. This resulted in participants in both groups with borderline scores approaching 24, which in turn resulted in a less pronounced pattern of variation in the results of pupillary response parameters ^18, 32 - 36^ . Pupillary responses D2 and LT also had a relatively small decline in function, although we can observe that the pattern of maximum pupillary constriction in the cognitively-impaired older adults group (D2 3.588 mm) was not as small as in the cognitively-unimpaired young adults group (D2 3.566 mm); in addition, the latency time before constriction starts is longer, which is LT 0.300 s compared to LT 0.267 s. This finding is similar to that of Fotiou et al. ^19^ , who showed that the dementia group had a 0.19 mm smaller mean D2 difference and 0.072 s shorter LT than the cognitively-unimpaired group. 

 To the best of our knowledge, there is no study whose authors analyzed the cognitive scoring using MMSE in relation to each pupillary response parameter. One of the most recent studies conducted by Chen et al., in 2022, linked pupillary response to a psychometric test using trail making test, modified Rey auditory verbal learning test, digit symbol substitution test, and verbal fluency test ^37^ . We found a significant relationship (p<0.05) between MMSE cognitive score and each pupillary response of D1, ΔD, CT, MCV, ACV, MCA, and ACA; however, there is no significant relationship between D2 and LT. These results are in line with the findings of Chen et al. ^37^ , but contradict the findings of Fotiou et al. ^19^ , who showed significant differences in LT and D2 scores between the cognitively-unimpaired and cognitively-impaired groups. We analyzed differences in the significance of these results, and the findings in relation to the study of Fotiou et al. ^19^ were:

- The sample number is relatively small, with 23 participants in each group of cognitively-unimpaired individuals and individuals with dementia;
- The study only covers dementia patients, not including MCI patients;
- The study indirectly compares the relationship between pupillary response parameters to a cognitive score of each group, only comparing the significance of the difference between LT and D2 in the cognitively-unimpaired and dementia groups.

Furthermore, there are differing findings in the literature regarding the significant relationship of LT with cognition. Similar to our findings, Prettyman et al.^38^ (n=9), Frost et al.^24^ (n=19), Bittner et al.^25^ (n=66 DA, and n=42 MCI), and Van Stavern et al.^39^ (n=24) also concluded that there was no significant relationship between LT and cognition; however, there was a significant difference in LT in the two studies by Fotiou et al.^18,  19^ (n=23 and n=48), and Ferrario et al.^22^ (n=20) ^18, 19 , 23- 27, 34 , 39^ . 

 Authors of current studies support changes in decreased pupillary response in cognitively-impairment participants and AD, although results vary with inconsistent values for each parameter ^19, 25 , 37, 38, 40 - 44^ . Nonetheless, the current findings have not shown the diagnostic value of pupillary response and cutoff points that can be used as guidelines or recommendations for detecting AD. Thus, our results make up for this shortcoming. We found that, based on the existing pupillary response parameters, the values of D1 and D2 as size parameters and MCV and ACV as velocity parameters have diagnostic values. The cognitively-unimpaired group has cutoff points ΔD 1.77 mm (Sv: 61.3%; Sp: 80%; PPV: 47.76%; NPV: 86.88%), MCV ≥4.43 mm/s (Sv: 70.4%; Sp: 58.5%; PPV: 46.93%; NPV: 78.48%), and ACV≥;3.12 mm/s (Sv: 70.4%; Sp: 55.0%; PPV: 45.83%; NPV 77.5%). Based on the sensitivities and specificities of each parameter, the positive likelihood ratios (LR+) were calculated as follows: ΔD had an LR+ of 3.07, MCV had an LR+ of 1.70, and ACV had an LR+ of 1.56. These LR+ values indicate that a positive test result (defined as an abnormal value above the normal cognitive cutoff) is approximately three times more likely to occur in a person with cognitive impairment for ΔD, 1.7 times more likely for MCV, and 1.56 times more likely for ACV. 

 Several participants were excluded during the data processing due to several factors affecting the pupil detection and pupillary responses measurement. Similar to Yoo et al., who found that eyeblink has a confounding effect on pupillometer measurement, and a blink-locked pupillary response (BPR) system could be a solution to maintain pupillary shape and size continuation before and after eyeblink^45^ . However, we found that the BPR system might, to some extent, affect changes in pupillary response with a greater shift along with more eyeblinks, and it could create an interpretation bias. We also found that patterned contact lenses, mature cataracts, and narrow eyelid openings that close half the pupil shape would interfere in the pupillary responses measurement; in line with the study conducted by Chen et al., participants who exhibited excessive artifacts in their pupillary response, incomplete pupil recording processes, or insufficient pupil data that could interfere with the interpretation of the pupillary response were excluded^37^ . 

 The entire young adult group is composed of participants who hold at least a bachelor’s degree or a specialization degree to elicit higher cognitive contrast with full MMSE score compared to the older adults group^46^ . Conversely, most older adults have a level of education of <9 years (50.78%), similar to the population study conducted in Indonesia, in which most older adults have low levels of education^47^ . The majority of older individuals had cognitive impairment of MCI or AD, with a percentage of 51.94% (n=77) among the older adults group. These findings are in line with the report of Yaffe et al.^48^ , who showed that the prevalence of the older adults with cognitive impairment in the USA is more than 40%, and also higher than the findings of Suriastini et al.^49^ in Yogyakarta, Indonesia, with a dementia prevalence of 20.1%. 

 Further research is necessary to address the limitations of this study, specifically by including a larger and more diverse population with distinct cognitive groupings, such as preclinical AD, MCI, and mild to severe AD, and by incorporating comparisons with other specific biomarkers, including the APOE-ε4 gene, amyloid-β deposition, and the Tau protein. Moreover, longitudinal prospective follow-up with multiple phases of data collection is necessary to track changes in pupillary responses alongside cognitive alterations in patients. 

 All in all, in this study we demonstrated a significant relationship between cognitive status and several pupillary response parameters, particularly ΔD, MCV, and ACV, which showed diagnostic potential. ΔD had LR+ of 3.07, indicating that a positive test result is approximately three times more likely to occur in individuals with cognitive impairment, while MCV and ACV had LR+ values of 1.70 and 1.56, respectively, suggesting a smaller, yet notable, likelihood of cognitive impairment with abnormal test results. These findings highlight the potential of pupillary response as a noninvasive tool for distinguishing normal cognitive function from cognitive impairment, especially among older adults. The findings support the possibility of its widespread use as an early screening of dementia to complete the limitation of cognitive psychometric tests or even as its potential replacement in the future. 

## ACKNOWLEDGEMENTS

 The authors would like to thank the Center of Health Research (Puslitkes), School of Medicine and Health Sciences, AJCUI; and the Atma Jaya Neuroscience and Cognitive Center (ANCC) for supporting this study. The authors also thank Ika Suswanti for her help in the processing of this article. 
